# Framing protocol optimization in oncological Patlak parametric imaging with *uKinetics*

**DOI:** 10.1186/s40658-023-00577-0

**Published:** 2023-09-12

**Authors:** Qing Ye, Hao Zeng, Yizhang Zhao, Weiguang Zhang, Yun Dong, Wei Fan, Yihuan Lu

**Affiliations:** 1 Shanghai United Imaging Healthcare Co., Ltd, Shanghai, China; 2 Sun Yat-Sen University Cancer Center, Guangzhou, China

**Keywords:** Patlak, *uKinetics*, Protocol optimization, Total-body PET

## Abstract

**Purpose:**

Total-body PET imaging with ultra-high sensitivity makes high-temporal-resolution framing protocols possible for the first time, which allows to capture rapid tracer dynamic changes. However, whether protocols with higher number of temporal frames can justify the efficacy with substantially added computation burden for clinical application remains unclear. We have developed a kinetic modeling software package (*uKinetics*) with the advantage of practical, fast, and automatic workflow for dynamic total-body studies. The aim of this work is to verify the *uKinetics* with PMOD and to perform framing protocol optimization for the oncological Patlak parametric imaging.

**Methods:**

Six different protocols with 100, 61, 48, 29, 19 and 12 temporal frames were applied to analyze 60-min dynamic ^18^F-FDG PET scans of 10 patients, respectively. Voxel-based Patlak analysis coupled with automatically extracted image-derived input function was applied to generate parametric images. Normal tissues and lesions were segmented manually or automatically to perform correlation analysis and Bland–Altman plots. Different protocols were compared with the protocol of 100 frames as reference.

**Results:**

Minor differences were found between *uKinetics* and PMOD in the Patlak parametric imaging. Compared with the protocol with 100 frames, the relative difference of the input function and quantitative kinetic parameters remained low for protocols with at least 29 frames, but increased for the protocols with 19 and 12 frames. Significant difference of lesion *K*_i_ values was found between the protocols with 100 frames and 12 frames.

**Conclusion:**

*uKinetics* was proved providing equivalent oncological Patlak parametric imaging comparing to PMOD. Minor differences were found between protocols with 100 and 29 frames, which indicated that 29-frame protocol is sufficient and efficient for the oncological ^18^F-FDG Patlak applications, and the protocols with more frames are not needed. The protocol with 19 frames yielded acceptable results, while that with 12 frames is not recommended.

**Supplementary Information:**

The online version contains supplementary material available at 10.1186/s40658-023-00577-0.

## Background

In the clinical positron emission tomography (PET) scans, standardized uptake value (SUV), a semi-quantitative metric, is widely used. However, SUV is often affected by human and biological factors, e.g. time of imaging and alterations in the blood pool activity [1]. The injection-to-acquisition time deviations from the standardized protocols occur frequently, which, in particular, increases the inter-subject and intra-subject variability in SUV measurements [2, 3]. Kinetic modeling avoids many of these factors and thus offers absolute quantification in PET imaging [4]. The application of kinetic modeling, such as *K*_i_ derived from Patlak analysis, has great clinical potential by providing more specific information than SUV in the oncological diagnosis and treatment assessment [5, 6].

In recent years, in addition to research study purposes, kinetic modeling has attracted increased attention in the *clinical* studies with the development of total-body PET systems [7]. The long axial field of view (AFOV) brings benefits of simultaneous total-body imaging as well as the greatly improved sensitivity [8, 9]. The ultra-high sensitivity makes it possible to perform dynamic scans with a high-temporal-resolution protocol for the first time [10–12]. For example, 60-min dynamic data were able to be binned into 187 dynamic frames, with one second as the shortest duration of the early frames [11]. In [12], the first 120-s data were divided into 100-ms temporal frames to investigate fast tracer dynamics and to perform cardiac motion tracking. Table 1 shows a list of articles regarding kinetic modeling using the uEXPLORER systems [10, 11, 13–24], the first total-body PET/CT scanners with 194-cm AFOV (United Imaging Healthcare, Shanghai, China). There, framing protocols with a large number of temporal frames were often used to capture all the tracer dynamic changes, and the frames with short durations are applied for the early peak of the fast tracer activity changes. This practice is helpful to perform accurate estimation of the image-derived input function (IDIF); however, it also substantially increases the computation burden due to the large number of frames, and the over sampling in time may be of little use for the tissues with activity concentration gently varying over time. Although optimization of scan protocols, i.e., decreasing the whole scan duration, has been extensively investigated in the whole-body and total-body dynamic imaging [23, 25, 26], the optimization of framing protocols was rarely mentioned.

**Table 1:** List of articles regarding kinetic modeling using the uEXPLORER systems (2019–2022)

Year	References	Scan duration	Number of frames	Framing protocols
2019	Zhang et al. [10]	60 min	187	60 × 1 s, 30 × 2 s, 20 × 3 s, 12 × 10 s, 50 × 30 s, and 15 × 120 s
2020	Zhang et al. [11]	60 min	187	60 × 1 s, 30 × 2 s, 20 × 3 s, 12 × 10 s, 50 × 30 s, and 15 × 120 s
2021	Feng et al. [13]	90 s	Not available	Not available
2021	Fu et al. [14]	Not available	Not available	Not available
2021	Li et al. [15]	60 min	66	30 × 2 s, 12 × 10 s, 6 × 30 s, 12 × 120 s, and 6 × 300 s
2021	Liu et al. [16]	60 min	60	36 × 5 s, and 24 × 180 s
2021	Liu et al. [17]	75 min	97	24 × 5 s, and 73 × 60 s
2021	Liu et al. [18]	60 min	60	36 × 5 s, and 24 × 180 s
2021	Wang et al. [19]	60 min	29	6 × 10 s, 2 × 30 s, 6 × 60 s, 5 × 120 s, 4 × 180 s, and 6 × 300 s
2021	Lan et al. [20]	Not available	Not available	Not available
2021	Qi et al. [21]	Not available	Not available	Not available
2021	Wang et al. [22]	Not available	Not available	Not available
2022	Wu et al. [23]	60 min	Not available	Not available
2022	Huang et al. [24]	60 min	98	50 × 2 s, 20 × 10 s, 10 × 30 s, 10 × 60 s, and 8 × 300 s

Most of the aforementioned uEXPLORER dynamic studies [10, 11, 13–24] used different framing protocols with different customized software, which is difficult for cross-center standardization and comparison. Therefore, it is of great interest to develop a convenient software for the large-cohort clinical studies to explore the efficacy of kinetic modeling, especially for the total-body dynamic PET imaging. A practical, fast, and automatic workflow is also required to enhance the ability to perform widespread evaluation.

To bridge the gap, we developed a software package (*uKinetics*, a commercial software of United Imaging Healthcare) to perform kinetic modeling on the uEXPLORER. *uKinetics* offers time activity curve (TAC)-based analysis and parametric imaging with multiple input function options, i.e., IDIF, population-based input function (PBIF), and user-defined input function. *uKinetics* supplies not only graphical analyses, e.g., Patlak, Logan, and RE plots, but also the common compartmental models, e.g., one-tissue, two-tissue, and two-tissue irreversible compartmental models, with or without time delay estimation. In addition, deep learning-based CT image segmentation was embedded to create an automated workflow.

In this study, we leverage *uKinetics* to evaluate the dynamic framing protocols with different number of temporal frames. Here, we focus on the ^18^F-FDG oncological Patlak applications. Careful verification of *uKinetics* is performed by comparing it to PMOD (version 4.3, PMOD Technologies Ltd., Zurich, Switzerland), a commercial software used for the biomedical image processing, analysis, and modeling. Various softwares exist for kinetic modeling and parametric imaging [7]. Among all, PMOD is one of the most widely distributed and contains all the most commonly used compartmental models for various applications. Lastly, framing recommendation for the ^18^F-FDG oncological Patlak studies performed on the uEXPLORER using *uKinetics* is offered.

## Methods

### Data acquisition

Ten participants underwent 0–60 min dynamic PET imaging with 0.10 ± 0.02 mCi/kg (3.53 ± 0.67 MBq/kg) ^18^F-FDG injection on the uEXPLORER PET/CT system at Sun Yat-sen University Cancer Center, Guangzhou, China. This study was approved by the ethics committee, and all subjects were provided informed consent for participation. Table 2 shows the diagnosis information of the participants.

**Table 2:** Participants’ information

Index	Gender	Age	Weight (kg)	Dose	Diagnosis
1	Male	59	59	4.8 mCi (176.9 MBq)	Lung cancer
2	Male	55	59	4.4 mCi (161.8 MBq)	Lung cancer
3	Male	63	64	5.0 mCi (185.9 MBq)	Lung cancer
4	Male	68	61	4.8 mCi (177.2 MBq)	Lung cancer
5	Female	56	60	7.6 mCi (281.2 MBq)	Cholangiocarcinoma
6	Female	38	62	6.8 mCi (251.6 MBq)	Hysteromyoma
7	Female	66	70	8.1 mCi (300.1 MBq)	Liver cancer
8	Male	63	57	6.0 mCi (222.3 MBq)	Liver cancer
9	Male	54	69	5.5 mCi (202.2 MBq)	Lung cancer
10	Male	64	58	6.1 mCi (224.5 MBq)	Lung cancer

### Framing protocol and image reconstruction

The dynamic PET images were reconstructed using the TOF + PSF OSEM algorithm with 3 iterations and 20 subsets. A three-dimensional Gaussian filter of 3 mm in full width at half maximum (FWHM) was used for noise suppression in the reconstructed dynamic image. The corrections for randoms, attenuation, scatter, normalization, decay, and dead time were applied. Image size of 150 × 150 × 673, and the voxel size of 4 × 4 × 2.886 mm^3^ were used. Through visually checking the 100 reconstructed frames, only minor motion, which was mainly respiratory motion, was found during the scans, and no patient motion correction was applied.

The following protocols with different number of temporal frames were compared and were referred to as P-100f, P-61f, P-48f, P-29f, P-19f, and P-12f:

- P-100f (100 frames): 30 frames × 1 s, 30 × 5 s, 10 × 12 s, 5 × 60 s, 25 × 120 s.
- P-61f: 30 × 2 s, 6 × 10 s, 8 × 30 s, 4 × 60 s, 5 × 120 s, 8 × 300 s.
- P-48f: 12 × 5 s, 6 × 10 s, 8 × 30 s, 8 × 60 s, 8 × 120 s, 6 × 300 s.
- P-29f: 6 × 10 s, 2 × 30 s, 6 × 60 s, 5 × 120 s, 4 × 180 s, 6 × 300 s.
- P-19f: 6 × 10 s, 3 × 180 s, 10 × 300 s.
- P-12f: 6 × 10 s, 1 × 540 s, 5 × 600 s.

### Kinetic modeling

A UIH software package (*uKinetics*) was applied to implement voxel-based kinetic modeling. Developed using C +  + programming language, * uKinetics* consists of three main modules: basic image display and processing including segmentation of regions of interest (ROIs), generation of input function, and ROI-based or voxel-based regression of kinetic models. In the segmentation module, descending aorta (with a user-defined radius), lung, and liver can be automatically segmented on the CT images. Threshold-based semiautomatic segmentation and manual segmentation are also supported for other ROIs on the CT or PET images. Multiple options, i.e. IDIF, PBIF, or user-defined input function, are offered in the processing of input function. In the regression module, users are able to select the time frames to be fitted, and the Levenberg–Marquardt algorithm is applied in the nonlinear fit of compartmental models. As a commercial software package, *uKinetics* is accessible to all the medical centers using uEXPLORER.

In this work, the input function was extracted from a cylindrical ROI with 4-mm transversal radius in the descending aorta. The centerline of the descending aorta was automatically segmented on the CT image using a deep learning network and was dilated to obtain the cylindrical ROI. No correction for partial volume effect was performed. Individual input function was generated for each framing protocol. The same input functions and dynamic images were used in PMOD. Patlak analysis (t* = 10 min [27]) according to Eq. 1 was performed for each framing protocol.

1 $$\frac{{C_{{\text{T}}} \left( t \right)}}{{C_{{\text{P}}} \left( t \right)}} = K_{{\text{i}}} \frac{{\int_{0}^{t} {C_{{\text{P}}} \left( \tau \right)d\tau } }}{{C_{{\text{P}}} \left( t \right)}} + {\text{intercept}}$$

where *C*_T_ and *C*_P_ denote the concentration of tissue and plasma, respectively. The macro kinetic parameter *K*_i_ represents the net uptake rate of tracer, and *intercept* represents the combination of blood volume and distribution volume of reversible compartment in an unknown fraction [28].

### Evaluation

For the quantitative evaluation, normal tissues including lung, liver, kidney, spleen, bone, muscle, gray matter, and white matter were segmented manually or automatically. Specifically, lung, liver, spleen, bone, and kidney were segmented automatically on the CT image; gray matter and white matter were segmented semiautomatically on the PET image according to the threshold; muscle and lesions were segmented manually. Overall, 29 lesions were manually segmented from the 10 patients based on the 55–60-min PET images. In total, 109 ROIs, i.e., organs and lesions, were analyzed for each framing protocol. The average values were calculated within each ROI on the parametric images. ROI-based correlation analysis and Bland–Altman plots were performed to compare *uKinetics* and PMOD. Quantitative differences were also calculated between different framing protocols. PMOD is regarded as the gold standard in the verification of *uKinetics*, and P-100f was used as the reference protocol when comparing different protocols. The absolute and relative differences between parameter_1_ and parameter_2_ are defined as Eq. 1 and Eq. 2, respectively.

2 $${\text{Absolute difference = }}\left| {{\text{parameter}}_{1} - {\text{parameter}}_{2} } \right|$$

3 $${\text{Relative difference}} = \frac{{{\text{parameter}}_{1} - {\text{parameter}}_{2} }}{{{\text{parameter}}_{2} }}$$

## Results

### Verification of parametric imaging in *uKinetics*

**Fig. 1:**
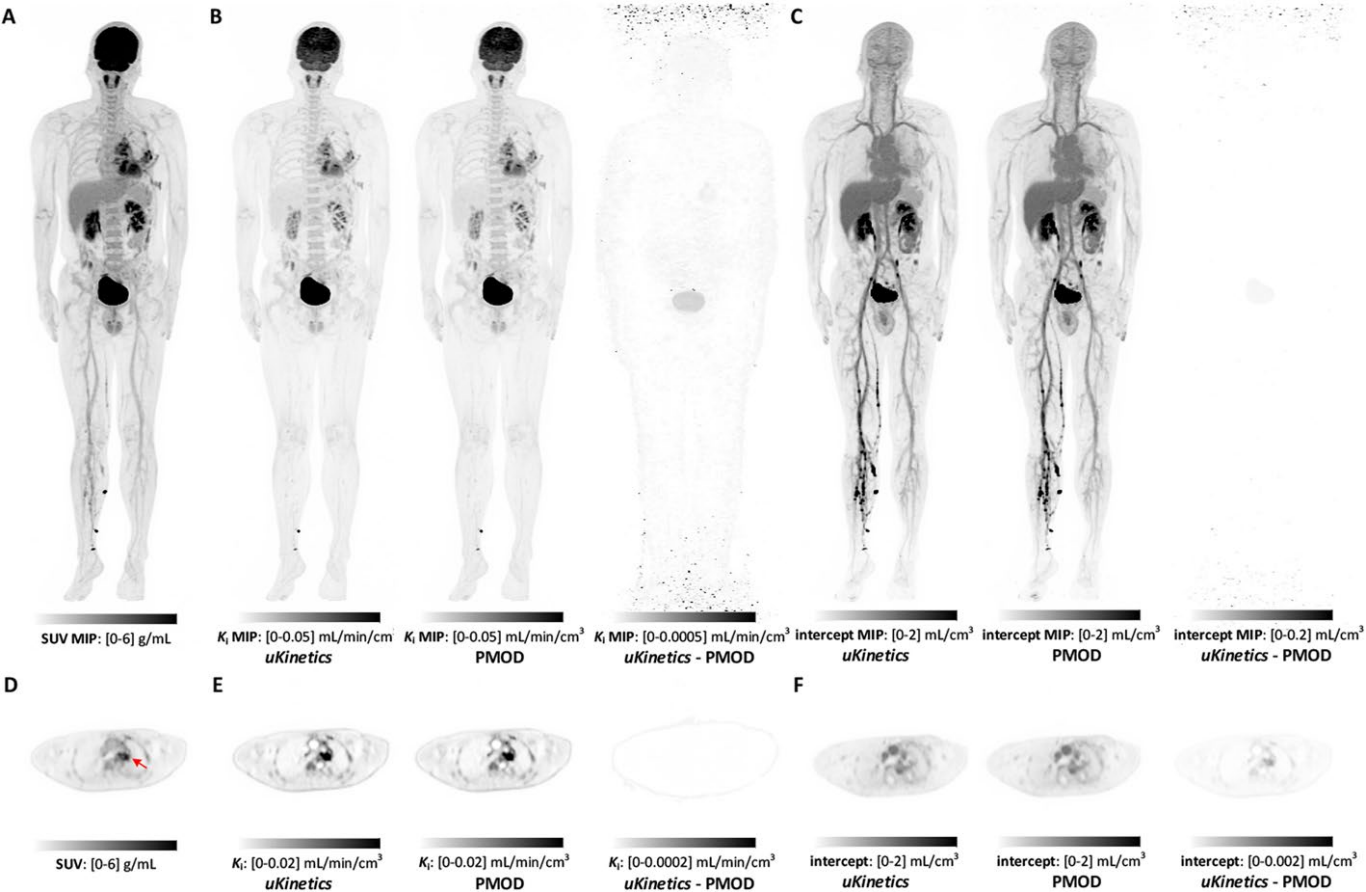
Comparison between *uKinetics* and PMOD for participant 1. **A** SUV (55–60 min) MIP image; **B** *K*_i_ MIP images; **C** intercept MIP images; **D** transverse view of the lesion (arrow) SUV (55–60 min); **E** transverse view of the lesion *K*_i_; and **F** transverse view of the lesion intercept. The parametric images were generated using *uKinetics* and PMOD, and absolute difference parametric images between them were also shown. The protocol of P-100f was used. Note: color scales are different for difference images from parametric images

Figure 1 shows SUV images, coronal maximum intensity projection (MIP), and transverse slices of the parametric images (P-100f) generated using *uKinetics* and PMOD for participant 1. The difference images between *uKinetics* and PMOD show the absolute maximum difference value in the anterior–posterior direction (as MIP), and the slice difference images show the absolute maximum difference in the transversal direction. Increased noise was shown at the edge of the axial FOV, which was due to the sensitivity drop toward the scanner edge. The difference was higher in the bladder region than other regions, which was likely due to the fact that the kinetic model was not suitable for the bladder. Overall negligible difference was observed between the parametric images generated from *uKinetics* and them from PMOD.

**Fig. 2:**
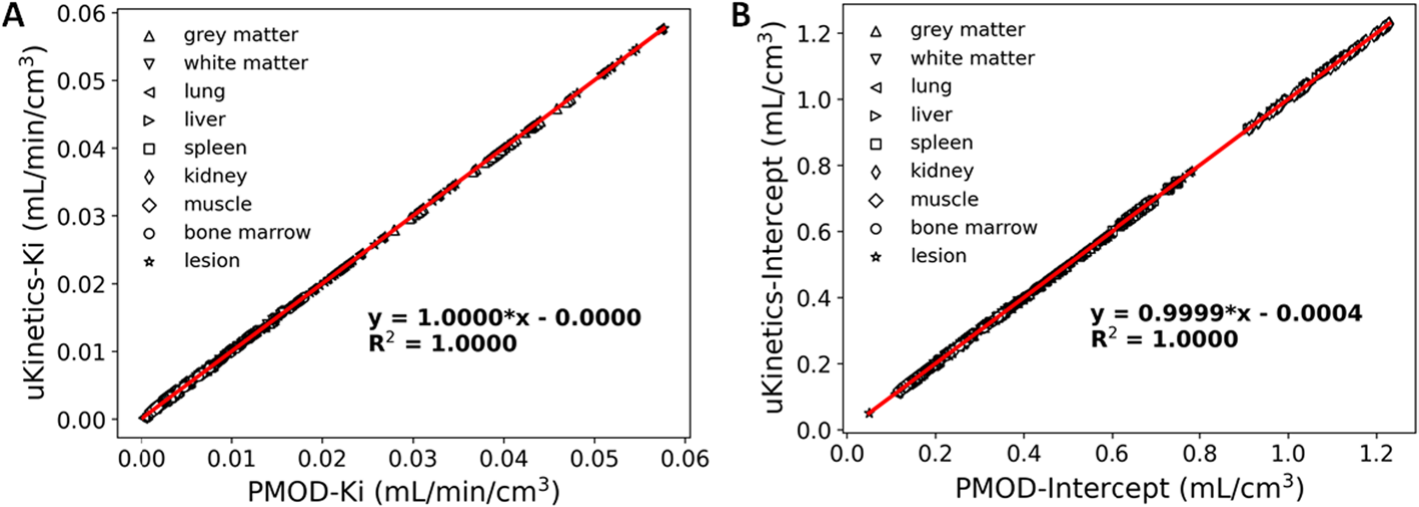
Correlation analysis of kinetic parameters: **A** *K*_i_ and **B** intercept between *uKinetics* and PMOD for all the ROIs (*N* = 109) and all the protocols (*N* = 6) of all the participants (*N* = 10)

**Fig. 3:**
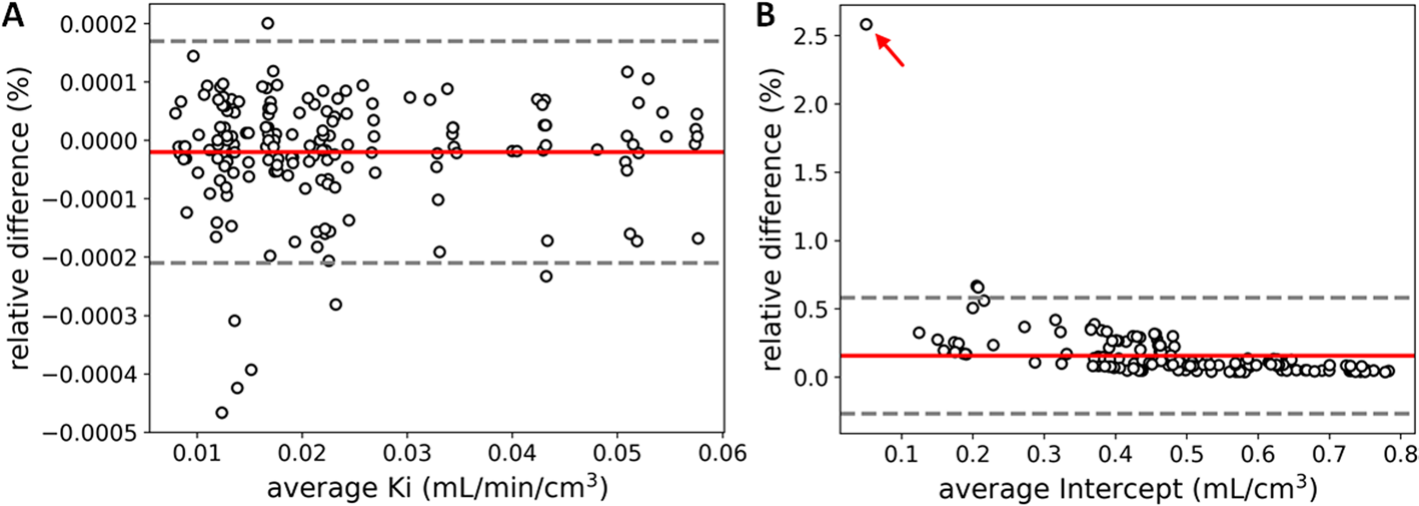
Bland–Altman plots of kinetic parameters: **A** *K*_i_ and **B** intercept between *uKinetics* and PMOD for all the lesions (*N* = 29) and all the protocols (*N* = 6) of all the participants (*N* = 10). An outlier was marked with an arrow

To quantitatively evaluate the kinetic parameters yielded by *uKinetics* and PMOD, correlation analysis was performed across all the 654 ROIs of the 10 subjects and 6 protocols. As shown in Fig. 2, excellent consistency (*R*^2^ > 0.9999) was found between *uKinetics* and PMOD for both *K*_i_ and intercept values. Figure 3 shows the Bland–Altman plots between *uKinetics* and PMOD for the lesions of all the subjects and protocols. Average relative difference was below 0.02% for both *K*_i_ and intercept, which proved excellent consistency between *uKinetics* and PMOD. For the outlier (red arrow) in Fig. 3B with > 2.5% relative difference, small absolute difference of 0.0013 mL/cm^3^ was found.

### Framing protocol optimization

**Fig. 4:**
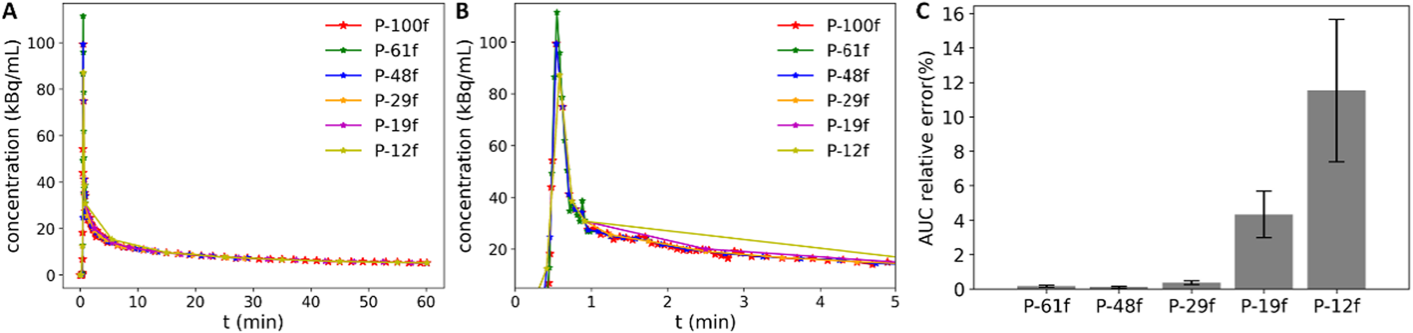
**A** IDIFs of participant 1 with different framing protocols; **B** Zoom-in of the 0–5 min input functions of A; **C** Mean ± SD of input function AUC relative error across the 10 patients, P-100f as reference. IDIF: image-derived input function. AUC: area under curve

Given the excellent consistency in parametric images found between *uKinetics* and PMOD, the Patlak analysis in *uKinetics* is regarded as verified. Here, we evaluated different framing protocols with *uKinetics*. IDIFs varied across different framing protocols, as shown in Fig. 4. The input functions showed different curve shapes especially in the early time. The area under the curve (AUC) values for the 0–60 min input function were calculated, and the mean ± SD values of the AUC relative error compared to P-100f across all the 10 patients were shown. Comparing to P-100f, the difference in the AUC values was below 0.4% for P-61f, P-48f, and P-29f, which was substantially higher for P-19f (4.3% ± 1.4%) and P-12f (11.5% ± 4.1%).

**Fig. 5:**
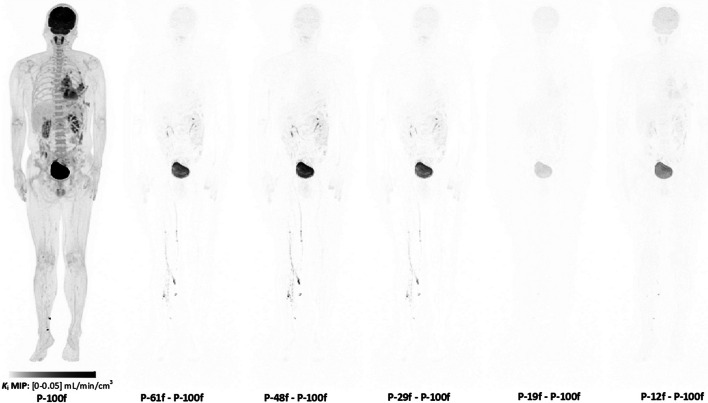
The *K*_i_ images of participant 1 with different temporal protocols

With the individual input function for each framing protocol, Fig. 5 shows the *K*_i_ MIP images of the participant 1 with the P-100f protocol, as well as the absolute difference images between other protocols and P-100f. Visually minor difference was observed in the *K*_i_ images between different protocols, except for the bladder region.

**Fig. 6:**
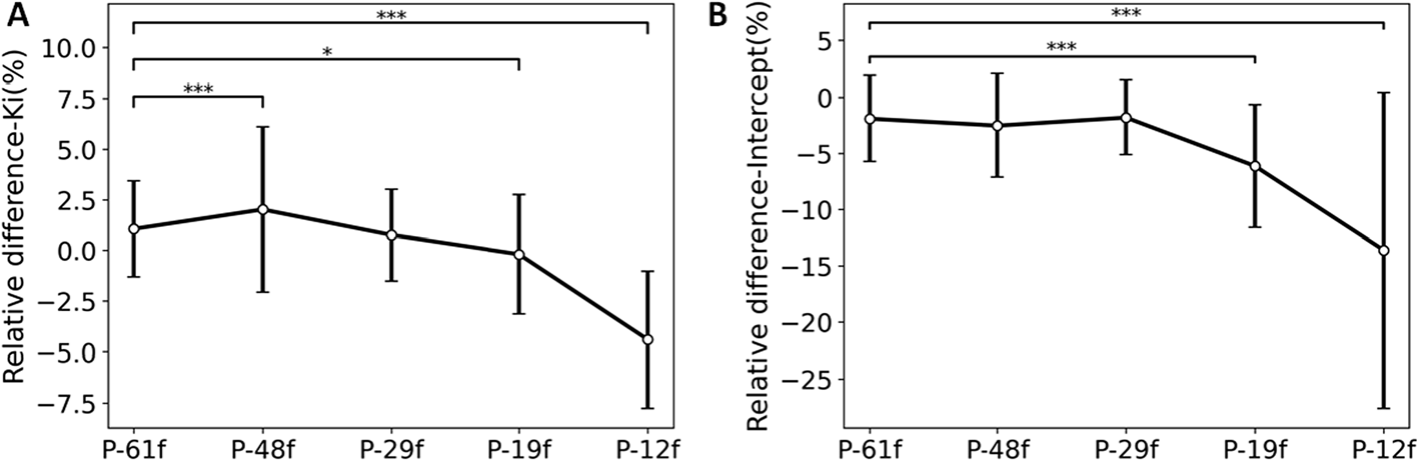
Mean ± SD relative difference of **A** *K*_i_ and **B** intercept values compared to P-100f (t* = 10 min) among the 10 participants. * indicates that the difference is significant (*: *p* value < 0.05, **: *p* value < 0.01, ***: *p* value < 0.001) with F-test

Table 3 shows the ROI-based quantitative *K*_i_ values for all the six protocols. Paired t-test was applied to the lesion *K*_i_ values and showed significant difference (*p* value < 0.05) between P-100f and P-12f. The relative difference of *K*_i_ and intercept values compared to P-100f is shown in Fig. 6. Largest average relative difference was found for *K*_i_ values with P-12f (-4.4%) and for intercept values with P-19f (-6.1%) and P-12f (-13.6%). Both *K*_i_ and intercept showed significant difference (*p* value < 0.001) with F-test on the variance for P-12f. Although significant difference was also observed between P-61f and P-48f in Fig. 6A, the variance of *K*_i_ relative difference was similar among all the protocols. The standard deviation was 2.4% and 4.1% for P-61f and P-48f, respectively.

**Table 3:** *K*_i_ values in the ROIs of difference framing protocols (mean ± SD of 10 subjects, unit: μL/min/cm^3^)

ROI	P-100f	P-61f	P-48f	P-29f	P-19f	P-12f
Lesion	23.5 ± 13.6^*^	23.6 ± 13.6	23.7 ± 13.8	23.5 ± 13.7	23.6 ± 13.9	22.4 ± 13.0^*^
Gray matter	36.5 ± 9.1	36.9 ± 9.5	37.2 ± 9.5	36.9 ± 9.5	36.2 ± 9.5	34.8 ± 9.3
White matter	11.6 ± 2.6	11.8 ± 2.7	11.9 ± 2.7	11.7 ± 2.7	11.0 ± 2.5	10.6 ± 2.5
Lung	0.9 ± 0.6	0.9 ± 0.6	0.9 ± 0.6	0.9 ± 0.6	0.9 ± 0.6	0.9 ± 0.5
Liver	3.7 ± 0.9	3.8 ± 1.0	3.8 ± 0.9	3.8 ± 1.0	3.8 ± 1.0	3.6 ± 0.9
Spleen	4.5 ± 1.8	4.6 ± 1.9	4.6 ± 1.9	4.6 ± 1.9	4.5 ± 1.9	4.3 ± 1.8
Bone marrow	9.8 ± 4.3	9.9 ± 4.4	9.9 ± 4.4	9.9 ± 4.4	9.6 ± 4.3	9.2 ± 4.1
Kidney	5.5 ± 2.6	5.5 ± 2.7	5.6 ± 2.7	5.5 ± 2.7	5.6 ± 2.6	5.3 ± 2.5
Muscle	1.6 ± 0.3	1.7 ± 0.4	1.7 ± 0.4	1.7 ± 0.4	1.6 ± 0.3	1.5 ± 0.3

*Significant difference (*p* value < 0.05) with paired t-test was found for lesion *K*_i_ values between P-100f and P-12f

**Fig. 7:**
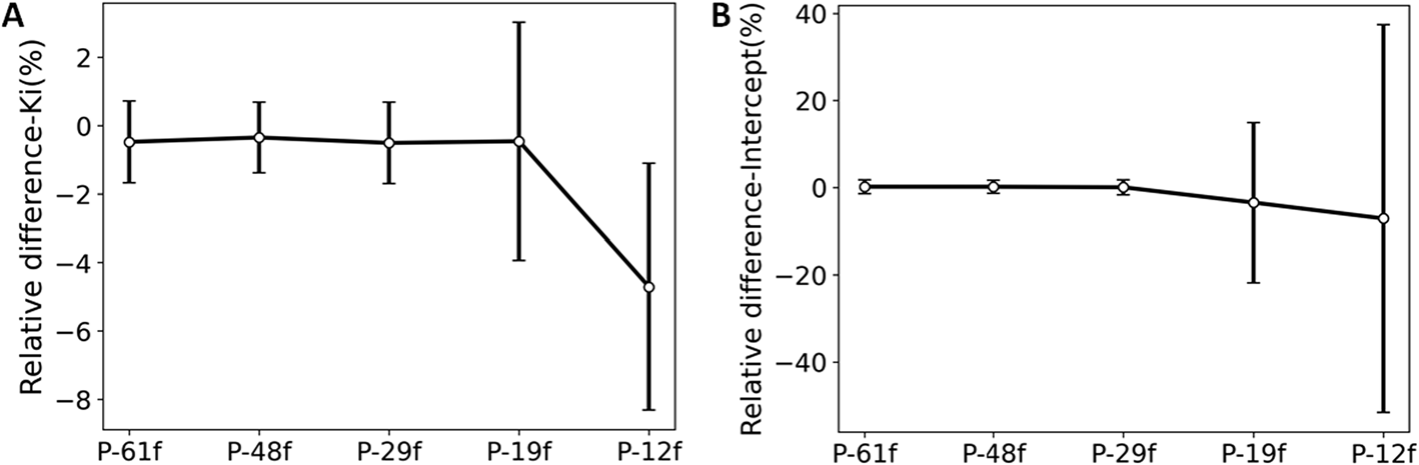
Mean ± SD relative difference of **A** *K*_i_ and **B** intercept values compared to P-100f (t* = 30 min) among the 10 participants

In the parametric imaging with Patlak model, 10–60 min data were used to generate *K*_i_ images. Later t* would also be used in clinical applications, because the optimized t* varies for different tissues. To evaluate the effects of t* on protocol optimization, the comparison of different framing protocols was also performed with t* = 30 min. As shown in Fig. 7, largest average relative difference was found for *K*_i_ values with P-12f (-4.7%) and for intercept values with P-19f (-3.5%) and P-12f (-7.1%).

## Discussion

With the quantitative comparison, *uKinetics* was proven to provide equivalent oncological FDG Patlak parametric imaging as compared to PMOD. One advantage of *uKinetics* is the automatic analysis procedure with the IDIF. With the user-defined radius of aorta ROI, AI-aided IDIF can be generated automatically. Since the software is available to all the medical centers using uEXPLORER, the fast and automated workflow will improve the clinical feasibility and standardization of dynamic PET, thus facilitating in the cross-center comparisons and collaborations. Besides, *uKinetics* is also applicable to the dynamic images acquired on other PET scanners, e.g., scanners with shorter AFOV than the uEXPLORER, when the descending aorta is within the FOV, although the robustness of the CT-based aorta segmentation is not guaranteed for CT scanners from other manufacturers.

In the optimization of framing protocols, P-29f showed similar results with P-100f, while P-12f showed significant difference. An important source of the parametric quantitative difference is the input function difference due to the sparse sampling with few temporal frames. The temporal framing protocol has a great effect on the IDIF. Interpolation of input functions to a framing protocol with dense sampling could reduce the error. If the input function was estimated by sufficient blood samples, the optimized number of frames could even be fewer. As for the P-19f, although the *K*_i_ values with P-19f did not show significant difference compared to those with P-100f as shown in Table 3, the differences of input function and intercept values were substantially high. The protocol with 19 frames may be acceptable but not recommended. It is interesting to note that P-19f yielded lower *K*_i_ difference when compared to P-100f than it with other protocols as shown in Figs. 5 and 6, which could be due to the uniform time frames used in the linear regression.

The impact of t* on the optimization of framing protocols was also evaluated. With the comparison between Fig. 6 and Fig. 7, the trend of relative difference for different protocols of t* = 30 min was consistent with that of t* = 10 min. Minor impact of t* was observed in the protocol optimization. When t* = 30 min, although the same sample points (6 × 300 s) were used for P-61f, P-48f, P-29f, and P-19f, the integral value of input function was different between P-19f and the other three protocols. Thus, P-19f showed similar *K*_i_ but different intercept, which was not recommended for the oncological Patlak analysis.

The main limitation in this work is that motion correction was not applied on the dynamic frames. Various types of motion could cause over- and under- estimation artifacts on the parametric images and mismatch between *K*_i_ and intercept images in Patlak analysis [29, 30]. Motion is more likely to occur during the total-body uEXPLORER dynamic scans, with the entire body in the entire FOV for a long scan duration [31]. A variety of studies have proved the feasibility and effectiveness of motion detection and correction algorithms for the dynamic PET studies, which can also be migrated to manage the motion effects during the dynamic total-body scans, especially the respiratory motion and inter-frame body motion [30–32]. Specifically, respiratory pattern variation in the time course would lead to difference in parameter estimation, which is an interesting topic for future research. The scans in this work were selected carefully, only including scans with minor motion, but small artifacts were still shown in the parametric images because of the mismatch between PET and CT. Nonetheless, the small artifacts due to motion were not expected to influence the conclusion in this paper, since the verification of *uKinetics* and optimization of protocols were performed for the same study population under the same condition.

Another limitation is that IDIF can only estimate the concentration in the whole blood rather than the plasma. However, the plasma-to-blood ratio is close to 1 for ^18^F-FDG [33, 34] and has little effect on the conclusion in this paper. Additionally, compartmental models to estimate the micro-parameters are also supported in *uKinetics*, and the verification and protocol optimization are going to be investigated in the future.

## Conclusion

This study presented the validation of the proposed kinetic modeling software (*uKinetics*) using PMOD and the optimization of framing protocols in the oncological Patlak applications. *uKinetics* provided equivalent oncological FDG Patlak indirect parametric imaging as compared to PMOD. Minor differences were found between protocols with 100 and 29 frames, which indicated that 29-frame protocol is sufficient and efficient for the oncological ^18^F-FDG Patlak applications, and the protocols with more frames are not needed. The protocol with 19 frames yielded acceptable results, while that with 12 frames is not recommended (Additional file 1).

### Supplementary Information

**Additional file 1: Fig. S1.** ROI delineation of participant 1.** Fig. S2.** Relative difference of kinetic parameters with the same input function applied to different framing protocols.** Fig. S3.** The sum squared error of Patlak parametric imaging with* uKinetics*.** Fig. S4.** Correlation analysis of kinetic parameters for all the voxels within ROIs.** Fig. S5.** Relative difference of kinetic parameters with the same input function and same number of frames applied to different framing protocols and** Fig. S6.** The interface display of* uKinetics*.

## Data Availability

The datasets used and analyzed during the current study are available from the corresponding author on reasonable request.

## Abbreviations

PET: Positron emission tomography
SUV: Standardized uptake value
AFOV: Axial field of view
IDIF: Image-derived input function
TAC: Time activity curve
PBIF: Population-based input function
FWHM: Full width at half maximum
ROI: Region of interest
MIP: Maximum intensity projection
AUC: Area under curve
