# Supplementary material for: Framing protocol optimization in oncological Patlak parametric imaging with uKinetics
Source: EJNMMI Phys. 2023 Sep 12;10:54. doi: 10.1186/s40658-023-00577-0 (PMC10497476; doi:10.1186/s40658-023-00577-0)
Supplement: Supplementary file 1 — Additional file 1: Fig. S1. ROI delineation of participant 1. Fig. S2. Relative difference of kinetic parameters with the same input function applied to different framing protocols. Fig. S3. The sum squared error of Patlak parametric imaging with uKinetics. Fig. S4. Correlation analysis of kinetic parameters for all the voxels within ROIs. Fig. S5. Relative difference of kinetic parameters with the same input function and same number of frames applied to different framing protocols and Fig. S6. The interface display of uKinetics. [file 40658_2023_577_MOESM1_ESM.docx]

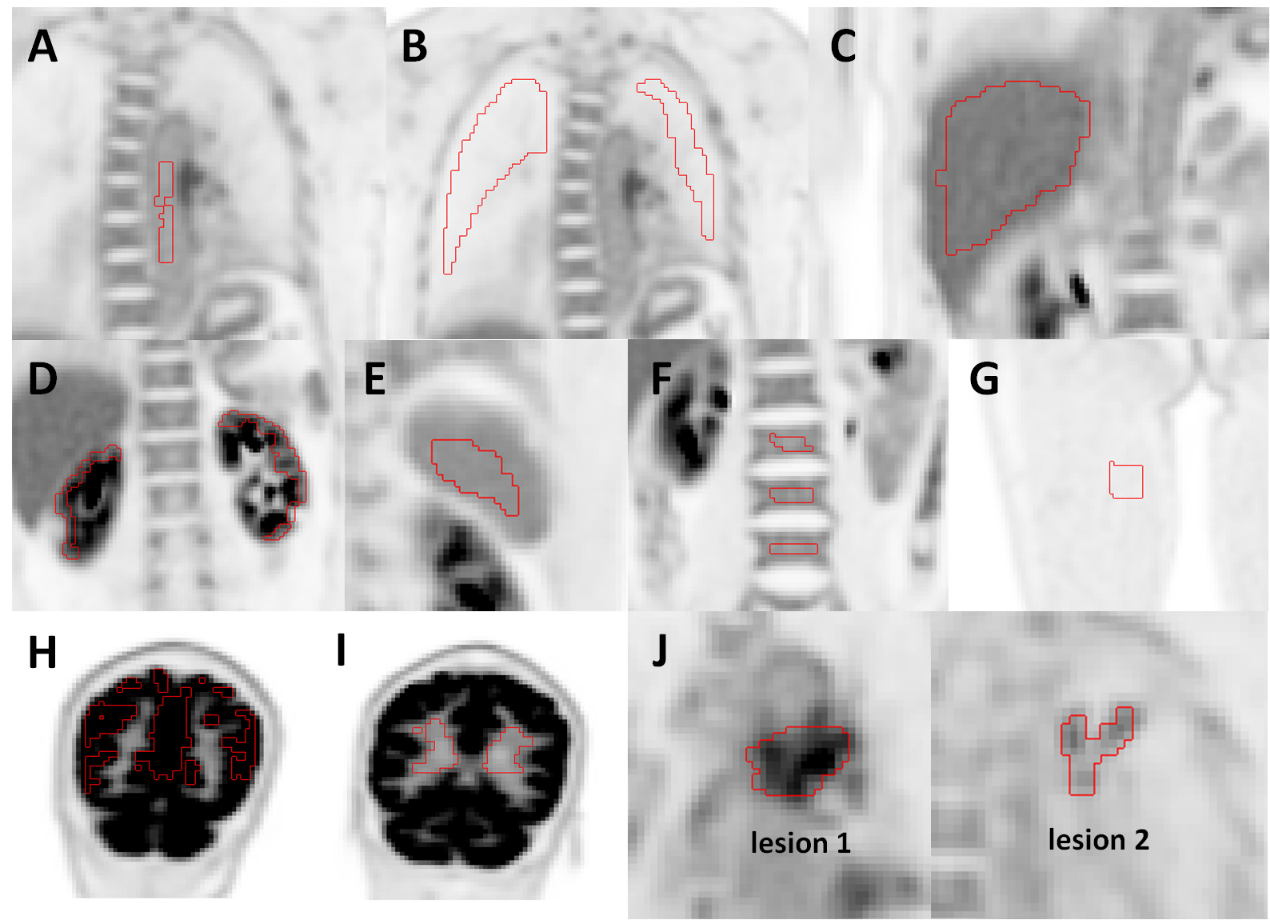


**Fig. S1** ROI delineation of participant 1: **A** aorta; **B** lung; **C** liver; **D** kidney; **E** spleen; **F** bone; **G** muscle; **H** grey matter; **I** white matter; **J** lesions.

To estimate the influence of input functions on the parametric images, the same input function from P-100f was applied to the other framing protocols. **Fig. S2** showed the relative difference of *K*_i_ and intercept values compared to P-100f. Compared to the results using individual input function for each protocol (Fig. 6 in the manuscript), the relative difference of *K*_i_ and intercept decreased, especially for P-12f. The source of the difference mainly came from the input function due to the sparse sampling of the protocol with few temporal frames.


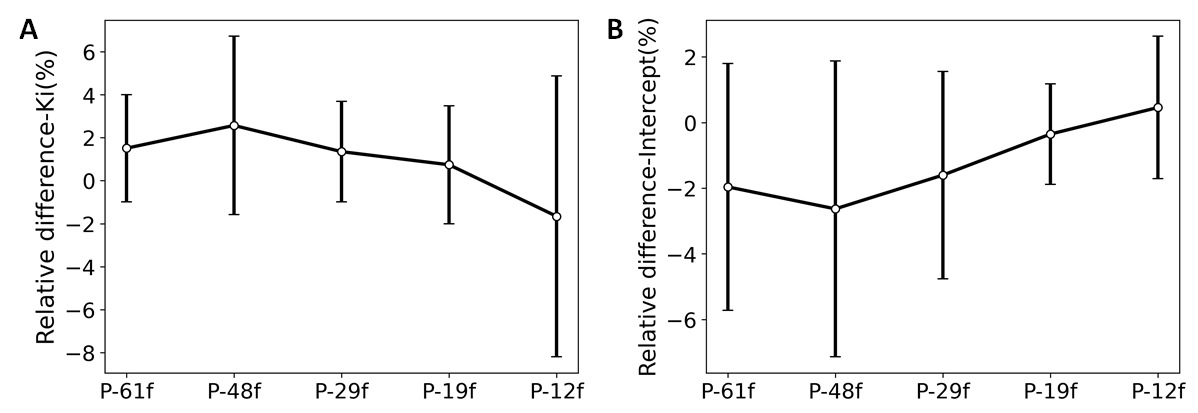


**Fig. S2** Mean ± SD relative difference of **A** *K*_i_ and **B** intercept values compared to P-100f (*t** = 10 min) among the 10 participants. The input function of P-100f was applied to the other protocols.

We calculated the sum squared error (SSE) to estimate the goodness of fit in the Patlak parametric imaging, which was defined as

$SSE=\sum_{t} \left(y\left(t\right)-\hat{y}\left(t\right)\right)^{2},t>t*$ (S1)

$y\left(t\right)=\frac{C_{\text{T}}\left(t\right)}{C_{\text{P}}\left(t\right)}$ (S2)

$\hat{y}\left(t\right)=\frac{\int_{0}^{t} C_{\text{P}}\left(\tau\right)d\tau}{C_{\text{P}}\left(t\right)}K_{\text{i}}+\text{intercept}$ (S3)

**Fig. S3** showed the maximum intensity projection (MIP) of SSE image of participant 1 and P-100f. The high SSE values in the bladder region indicated the inadaptability of Patlak model.


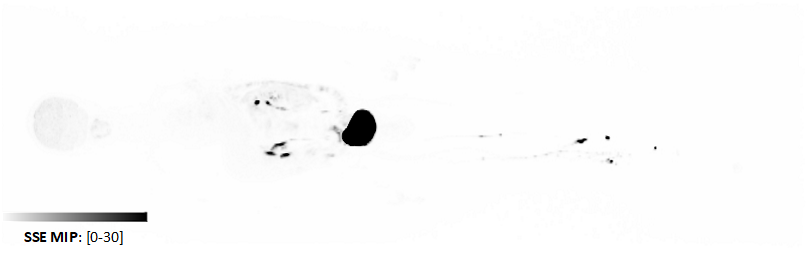


**Fig. S3** SSE MIP of Patlak parametric imaging of participant 1 and P-100f with *uKinetics*.


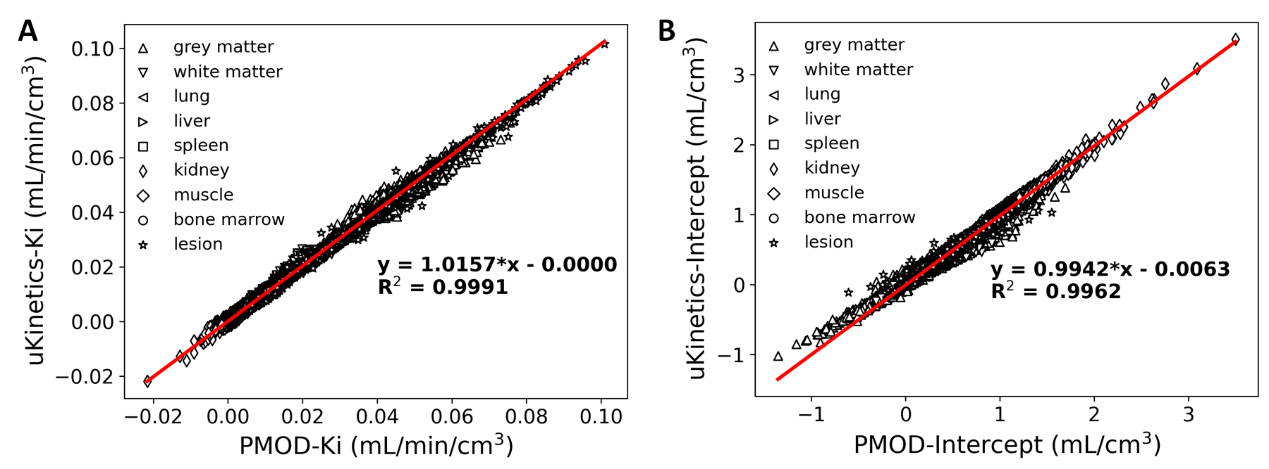


**Fig. S4** Correlation analysis of kinetic parameters: **A** *K*i and **B** intercept between *uKinetics* and PMOD for all the voxels (N = 322,998) within ROIs (N = 109) and all the protocols (N = 6) of all the participants (N = 10).

**Fig. S5** Mean ± SD relative difference of **A** *K*_i_ and **B** intercept values compared to P-100f (*t** = 10 min) among the 10 participants. The input function of P-100f was applied to the other protocols, and the same frame number of 5 in the post-*t** period was used.


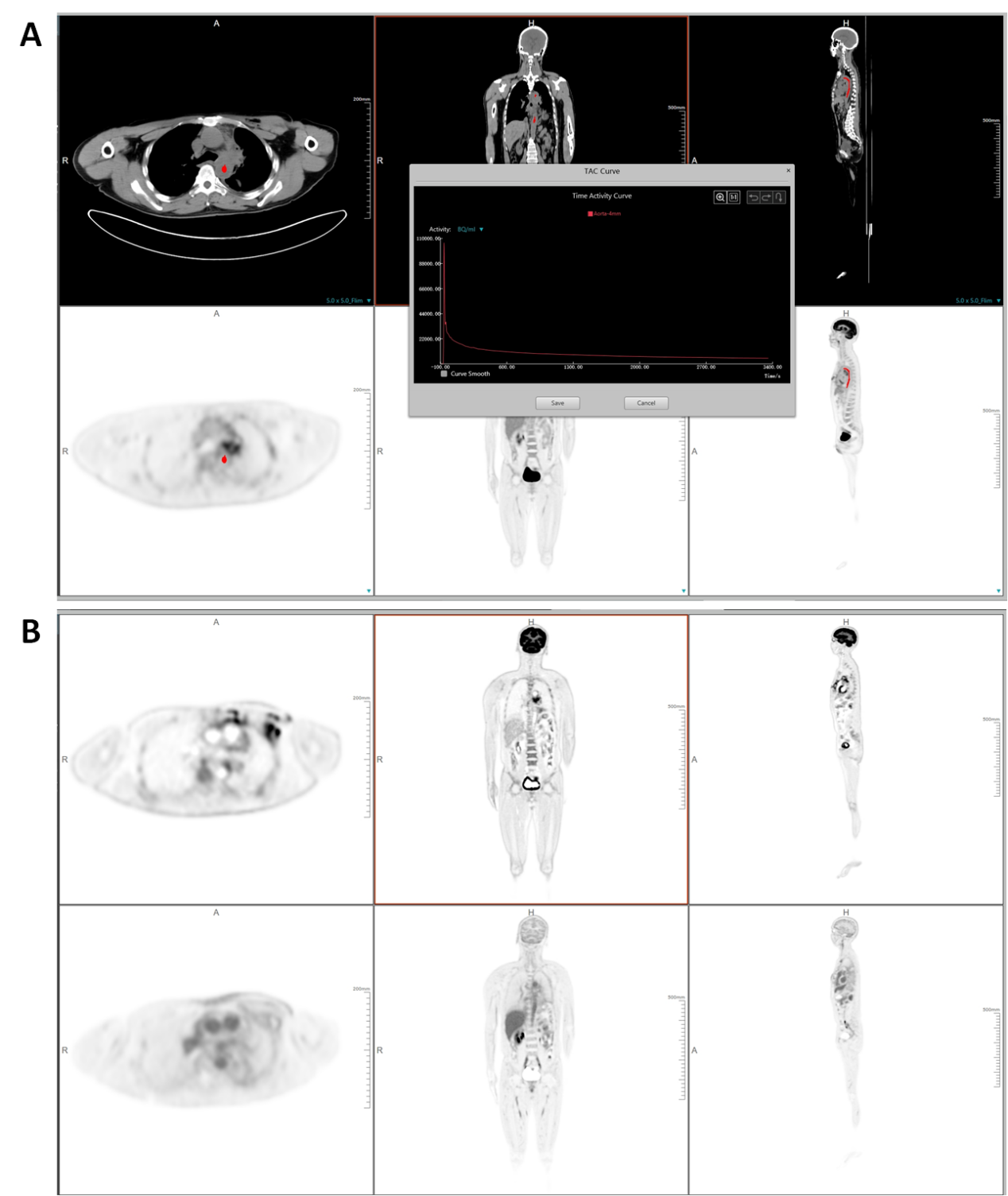


**Fig. S6** Interface of *uKinetics*: **A** display of image-derived input function (first row: CT image, second row: PET image), **B** display of Patlak parametric images (first row: *K*_i_ image, second row: intercept image).
